# Costs and health-related quality of life in relation to caries

**DOI:** 10.1186/s12903-019-0874-6

**Published:** 2019-08-16

**Authors:** Lisa Kastenbom, Alexandra Falsen, Pernilla Larsson, Karin Sunnegårdh-Grönberg, Thomas Davidson

**Affiliations:** 1 Department of Odontology, University of Umeå, Umeå, Sweden; 2 Department of Prosthodontics, Faculty of Odontology, Malmö University, Malmö, Sweden; 3 Centre of Oral Rehabilitation, Folktandvården Östergötland, Norrköping, Sweden; 4 Centre for Medical Technology Assessment (CMT), Department of Medical and Health Sciences, (IMH), Linköping University, SE-581 83 Linköping, Sweden

**Keywords:** Caries, Health economics, Quality of life, Costs

## Abstract

**Background:**

Dental caries remains a common and expensive disease for both society and affected individuals. Furthermore, caries often affect individuals’ health-related quality of life (HRQoL). Health economic evaluations are needed to understand how to efficiently distribute dental care resources. This study aims to evaluate treatment costs and QALY weights for caries active and inactive adult individuals, and to test whether the generic instrument EQ-5D-5 L can distinguish differences in this population.

**Methods:**

A total of 1200 randomly selected individuals from dental clinics in Västerbotten County, Sweden, were invited to participate. Of these, 79 caries active and 179 caries inactive patients agreed to participate (response rate of 21.7%). Inclusion criteria were participants between 20 and 65 years old and same caries risk group categorization in two consecutive check-ups between 2014 and 2017.

**Results:**

Treatment costs showed to be twice as high in the caries active group compared to the caries inactive group and were three times higher in the caries active age group 20–29 compared to the caries inactive age group 20–29. Differences between the groups was found for number of intact teeth according to age groups. In the EQ-5D-5 L instrument, more problems relating to the dimension anxiety/depression was seen in the caries active group. QALY weights showed tendencies (non-significant) to be lower in the caries active group.

**Conclusions:**

These findings highlight the need for efficient treatments and prevention strategies as well as adequate money allocation within dentistry. However, further research is needed to assess appropriate instruments for health economic evaluations.

## Background

Dental diseases have a considerable global economic impact: in 2010 direct treatment corresponded to 4.6% of global health costs [1]. In Sweden, the annual total spending on dental care is about 2.5 billion Euro [2] (about 245 Euro per capita). In most countries, untreated dental caries is a major challenge for public health. In 2010, untreated dental caries was the most prevalent condition worldwide for both adults and children [3]. As health care should be regarded in a context of finite resources, decision-makers need to evaluate health economics to choose the most cost effective prevention and treatment programs [4]. In the future, information about economic evaluation in dentistry is likely to be required for resource allocation. Despite the above knowledge, economic evaluations are rarely used in dentistry [5–7]. Quality of Life (QoL) assessments have been introduced to support and improve decisions within health care as well as for ethical reasons. To ease the comparability of measurements, Health-Related Quality of Life (HRQoL) has been introduced as an indicator of an individual’s well-being. HRQoL describes the impact a specific disease has on an individual’s QoL. In the last decades, interest has increased for studying the experiences of Oral Health-Related Quality of Life (OHRQoL). OHRQoL considers individuals’ perspectives regarding oral health and what impact oral health has on their everyday well-being. Previous literature has shown that presence of caries impacts a person’s OHRQoL [8, 9]. One of the instruments frequently used to assess OHRQoL is the Oral Health Impact Profile (OHIP) [10].

To assess the value of QoL over time, the concept quality-adjusted life-years (QALYs) is often used and this measure is generally recommended as an outcome measure in health economic evaluations [11]. QALYs are commonly used to evaluate health care and estimate the impact of a certain health state or treatment [4]. QALY measures both life span and HRQoL, where the latter is measured with QALY weights. QALYs are calculated as the QALY weight multiplied by the time in a specific health state. The QALY weight is scored between 0 (death) and 1 (full health) and can be measured by direct methods such as standard gamble (SG), time trade-off (TTO), and visual analogue scale (VAS). Another way to measure QALY weights is to use an indirect method such as generic questionnaires (e.g., EQ-5D or SF-6D) [12]. The responses in the questionnaires are paired to an evaluation system, a tariff/value set, or an algorithm. To measure the potential effects of new dental technologies and treatment strategies on QoL, two types of instruments can be used: a generic instrument such as the EQ-5D or a disease-specific instrument such as OHIP [5, 13]. Developed by EuroQoL, EQ-5D is a standardized generic instrument used to describe and evaluate HRQoL [14–16]. The instrument is a self-reported questionnaire that consists of a descriptive part with five dimensions of health and a VAS scale [17]. Each of the five dimensions can be answered in the original three level (3 L) or a newer extended five level version (5 L). Research funded by the EuroQol found that the 5 L version catches more nuance and reduces ceiling effects [18]. Brennan and Spencer have attempted to map OHIP to EQ-5D [19], but no well-accepted method for deriving QALY from OHIP has yet been developed.

This study has two aims: (i) to evaluate treatment costs and QALY weights for caries active and inactive individuals and (ii) to test whether EQ-5D-5 L can distinguish OHRQoL differences in an adult population.

## Methods

This study is part of a larger project about caries treatment in adults (to be reported elsewhere) being conducted in Västerbotten County, Sweden. The Regional Ethics Review Board in Umeå reviewed and approved the study protocol (Dnr: 2017/349–32).

### Population and study design

Patients from Public Dental Health Service clinics in Västerbotten County were invited to participate in the study. All Public Dental Health clinics except those situated in the Umeå region took part since the clinics in this region had previously contributed to a pilot study. Inclusion criteria were patients between 20 and 65 years of age 1 October, 2017 and who had been to dental check-ups twice during the years 2014–2017. Patients regularly visiting the clinics for their dental treatment are categorized into three different risk groups due to their general and dental status. No caries activity is referred to as “no/low caries risk” and several new caries lesions as “high caries risk” (Table 1). Between these two risk groups, there is a risk group categorized as “moderate risk”, but this group is not included in this study. In 2014, dental records revealed that 35,178 individuals had a caries risk assessment. All patients who had no/low caries risk at dental check-ups twice during the years 2014–2017 formed the caries inactive group (CI) and all patients who had high caries risk twice during the same period of time formed the caries active group (CA) in this current study. The CI group consisted of 5736 and the CA group consisted of 1254 individuals. After random selection, 600 individuals in each group were finally invited to participate in the study. In January 2018, these individuals were mailed written information about the study, a questionnaire, and a pre-stamped return envelope. No reminders were mailed. In total, 260 (21.7%) choose to participate – 179 (29.8%) from the CI group and 81 (13.5%) from the CA group. For all participants, data such as sex, age, and dental status were retrieved from computerized dental records. In addition, complementary information about number of visits, type of personnel seen at the visit, self-reported medical condition and medication, use of tobacco, type of treatment, and costs of treatment were retrieved from dental records. Three questionnaires were excluded due to internal failure.

**Table 1:** Overview of risk categories and criteria for risk assessment used in the Västerbotten County, Sweden

Risk category	Risk group 0 (no/low risk)	Risk group 1 (moderate risk)	Risk group 2 (high risk)
General	• No disease or medication affecting teeth or gums • Good oral hygiene • Adequate diet and intake frequency	• Disease and/or medication with possible effect on teeth or gums • Mediocre oral hygiene • Partly inadequate diet • Moderate dental anxiety • Smoker or snuff user	• Disease or medication with significant effect on teeth and gums • Poor oral hygiene • Inadequate diet • Severe dental anxiety • Heavy smoker (> 20 cigarettes/day)
Caries	• No active enamel or dentin caries lesions	• 1–2 new caries lesions on caries prone surfaces • New or moderate progression of enamel lesions	• ≥3 new caries lesions • Extensive progression of several enamel lesions • Lesions on non caries-prone surfaces
Periodontal	• Periodontal health • Gingivitis and/or supragingival Calculus • Bleeding-free gingiva and no pocket exceeding > 5 mm	• Periodontitis experience • Localized periodontal problems/signs of local bone loss • Bleeding and pocket depth of 5–6 mm	• Active periodontal disease with clinical radiographic attachment loss • Subgingival calculus • Peri-implantitis
Technical	• Intact teeth or few restorations • Single root canal treatment of good quality • Single crown or short bridge of good quality • No or minimal abrasion of teeth	• Single large restoration • Single restoration extending close to the pulp • > 1 root canal treatment of good quality • Erupting wisdom tooth in the lower jaw • Tongue/lip piercing • Moderate abrasion of teeth/TMD pain • Crowns and/or bridges on healthy teeth with good occlusion • Full or partial denture	• Several large restorations • Several root canal treatments or root canal treatments of inadequate quality • Wisdom tooth requiring surgery • Tooth grinding/TMD pain • Extensive erosion • Tongue or lip piercing with damaged teeth or mucosa • Extensive teeth or implant supported constructions

### EQ-5D-5 l

The EQ-5D-5 L questionnaire addresses five dimensions of health: mobility, self-care, usual activities, pain/discomfort, and anxiety/depression [17]. Each dimension has five answer options: no problems [1]; slight problems [2]; moderate problems [3]; severe problems [4]; and extreme problems [5]. At the end of the questionnaire, the participants were asked to rate their individual health today on a VAS from 0 to 100, where 0 is the worst health imagined and 100 the best health imagined. Questions without answers or more than one answer were excluded.

### QALY weight calculation

Health profiles were extracted from the answers in EQ-5D-5 L. The QALY weights were then calculated using a country (UK) specific value, translating the profile to an index value between zero (death) and one (perfect health) [20, 21]. For the EQ-5D-5 L, there is no country specific QALY value set for Sweden, so the UK version was used. The QALY weights were calculated in three ways. The first method used the crosswalk between EQ-5D-5 L to EQ-5D-3 L [22, 23], the second method used the direct conversion tariff for EQ-5D-5 L established by Devlin [20], and the third method used the VAS in the questionnaire.

### Treatment costs

The total treatment cost for each individual and the mean cost for each risk group between 2014 and 2017 were calculated using reference rates gathered from The Dental and Pharmaceutical Benefits Agency (TLV) in Sweden. Reference rates are connected with specific treatment measures comprising all dental care performed. Reference rates also include costs for staff, materials, overhead, and development [24]. The individual care provider sets the price for each treatment, but subsidies provided by the Swedish Social Insurance Agency are based on the reference rates from TLV. General subsidy for adults, corresponding to reference prices, is applied according to a “high-cost” threshold. In this study, current reference rates between 2017 and 2018 were used to calculate costs except in cases with redefined treatment measures from 2014, where reference rates from 2013 and 2014 were used. The costs were measured in Swedish Krona (SEK) and presented in Euros using the exchange rate of SEK1 = €0.095, the exchange rate on February 10, 2019.

### Statistics

IBM SPSS Statistics 25 and Excel 2016 were used for statistic calculations. A *p*-value below 0.05 was defined as a statistically significant difference. The percentage of each answered option per domain and the average QALY weights were compared between the CA and CI group. For comparison between the CA and CI group and male and female, the Independent Samples T-test was used. Analysis of variance (ANOVA) was used for comparison between age groups.

## Results

### Respondents’ characteristics

The CA and CI groups differed in age and tobacco use, but not in sex, living area, general health status, or medication use. Compared to the CI group, the CA group received more advice about basic prevention, received more fluoride supplementation, and were given individual hygiene instructions. Diet counselling was given to one-quarter of the CA group. Approximately one-third of the CA group received follow-up on prevention advice. In an analysis of the non-responders a skewed distribution was seen between the sexes: women were more represented among the responders and men more represented among the non-responders irrespective of caries risk group. The CA group were significantly younger than the CI group for both the responders and non-responders. Statistically significant differences were found between the CI and the CA non-responders in DMFT, DMFS, and number of intact teeth, but not for total number of teeth. Table 2 lists respondents’ characteristics.

**Table 2:** Respondents’ characteristics

Variables	Caries inactive (*n* = 179)	Caries active (*n* = 81)	*P*-value
Accommodation area (%)			0.164
City	36	27	
Coastal Areas	20	23	
Rural Areas	44	50	
Age (years)	45.2	35.1	< 0.001
Gender (%)			0.073
Male	35.2	46.9	
Female	64.8	53.1	
Health status (%)			0.773
Healthy	70	72	
Diseased	30	28	
Medication (%)			0.932
Non medicated	60.2	62.0	
1–2 medicines	25.6	24.1	
> 3 medicines	14.2	13.9	
Tobacco use (%)			0.012
No tobacco use	88.7	77.2	
Present smoker	3.4	5.1	
Present snuff user	7.9	16.5	
Present smoker and snuff user	0	1.2	
Preventive/Non-operative measures (%)			
Basic prevention	50.8	86.1	< 0.001
Additions Fluoride	15.8	85.7	< 0.001
Individual counselling on oral hygiene	29.4	77.2	< 0.001
Individual counselling on diet	2.3	22.8	< 0.001
Follow-up on prevention advice	6.8	35.4	< 0.001

Compared to the CI group, the CA group had fewer intact teeth. In the CA age group the mean number of intact teeth distinctly reduced with each ten-year period. The CA group had more decayed teeth, irrespective of age group (Table 3).

**Table 3:** Dental and caries status in the CI and CA groups divided by age

Age intervals	20–29			30–39			40–49			50–59		
	CI *n* = 38	CA *n* = 31	*p*-value	CI *n* = 17	CA *n* = 26	*p*-value	CI *n* = 34	CA *n* = 13	*p*-value	CI *n* = 90	CA *n* = 11	*p*-value
Total number of teeth	29.08	29.87	0.121	29.29	28.19	0.124	28.65	28.15	0.522	27.59	25.64	0.008
Number of intact teeth	27.89	22.55	< 0.001	25.24	18.69	< 0.001	21.47	14.46	< 0.001	14.44	9.36	0.010
Caries Status												
DMFT	4.11	9.45	< 0.001	6.67	13.31	< 0.001	10.53	17.54	< 0.001	17.56	22.64	0.010
DMFS	16.08	24.13	0.015	20.76	38.04	0.012	29.21	49.31	< 0.001	50.06	79.91	< 0.001
DMFSa	6.16	10.48	0.002	8.41	15.73	0.027	11.18	21.54	< 0.001	20.97	35.45	< 0.001
DT	0.03	2.26	< 0.001	0.06	1.19	0.018	0.03	1.00	< 0.001	0.03	1.91	< 0.001
FT	1.16	6.26	< 0.001	4.00	9.15	< 0.001	7.18	13.54	< 0.001	13.13	16.18	0.078
DFT	1.18	7.32	< 0.001	4.06	9.50	< 0.001	7.18	13.69	< 0.001	13.13	16.27	0.070
DFS	1.53	13.48	< 0.001	7.35	19.15	0.013	12.47	30.08	< 0.001	28.08	48.27	< 0.001
DFSa	0.32	6.23	< 0.001	3.00	8.12	0.040	4.47	13.85	< 0.001	12.14	22.73	0.001
DS2	0.00	6.35	< 0.001	0.06	5.23	< 0.001	0.21	3.46	< 0.001	0.00	2.00	< 0.001
DSa2	0.00	5.71	< 0.001	0.00	4.77	< 0.001	0.21	3.31	< 0.001	0.00	1.55	< 0.001
DS3	0.03	2.03	< 0.001	0.06	1.15	0.014	0.00	0.54	0.002	0.01	0.36	< 0.001
DSa3	0.00	1.35	< 0.001	0.00	0.69	0.025	0.00	0.38	0.001	0.01	0.18	0.001
Secondary caries lesions	0.00	0.55	0.025	0.00	0.19	0.171	0.03	0.62	0.018	0.02	2.09	< 0.001
Secondary caries lesions a	0.00	0.42	0.027	0.00	0.08	0.425	0.00	0.46	0.019	0.02	1.27	< 0.001
Caries lesions (total)	0.03	2.55	< 0.001	0.06	1.35	0.017	0.03	1.15	< 0.001	0.03	2.45	< 0.001
Approximal caries lesions (total)	0.00	1.77	< 0.001	0.00	0.77	0.015	0.0	0.85	0.001	0.03	1.45	< 0.001

*D* decayed, *M* missed, *F* filled, *T* teeth, *S* surfaces, *a* approximal, *D2* decay to enamel and dentin junction, *D3* decay in dentin

## Resource use

**Fig. 1:**
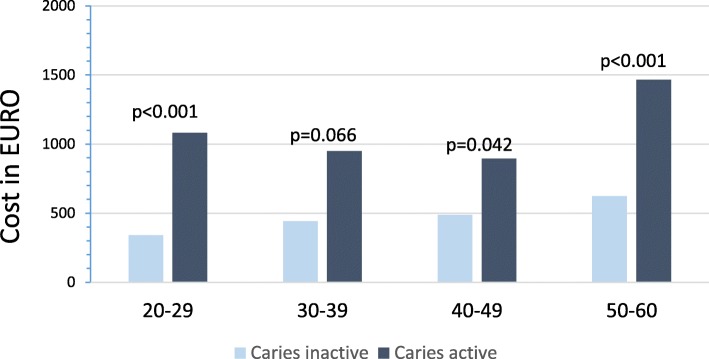
Mean treatment cost in the CI and CA groups divided by age

The CA individuals visited a dentist twice as often as the CI group, and on average, the CA individuals also visited a dentist twice as often as they visited a dental hygienist. The CA group had more visits and the recall interval was shorter than for the CI group. The mean treatment cost for each individual between 2014 and 2017 was nearly two times higher for the CA group compared to the CI group (Table 4). The mean treatment cost showed a tendency to increase with age in the CI group and there was a significantly statistical difference between the ages 20–39 and 40–60. In the CA group, no differences in treatment cost was found regarding age group (Fig. 1). The mean treatment cost were higher in the CA group regardless of age (*p* < 0.05) except between the ages 30–39, with the biggest difference for individuals in age group 20–29 with more than three times the cost.

**Table 4:** Resource use over the study period in the CI and CA groups

Resource use	Mean		*p*-value
	CI	CA	
Number of visits to dental clinic	5.20	9.52	< 0.001
Number of visits to dentist	3.06	6.36	< 0.001
Number of visits to dental hygienist	2.11	3.06	0.004
Number of acute visits to the clinic	0.86	1.01	0.455
Recall interval (months)	23.82	18.38	< 0.001
Mean cost per individual (EURO)	520	1062	< 0.001

### EQ-5D-5 l

The CA group tended to have a higher mean in all dimensions – i.e., this group experienced more problems. The CA group experienced more problems regarding anxiety/depression than the CI group and the difference was statistically significant. No difference was found in the other dimensions. Table 5 shows the answer percentage for each alternative.

**Table 5:** Percentage of answer on each question and risk group from EQ-5D-5 L questionnaire. P-value is calculated as independent sample t-test for mean value of CI vs CA

Dimensions	No problems		Slight problems		Moderate problems		Severe problems		Extreme problems		*p*-value
	CI	CA	CI	CA	CI	CA	CI	CA	CI	CA	
Mobility (%)	85.2	85.9	10.1	5.1	3.6	6.4	1.2	1.3	0	1.3	0.467
Self-care (%)	95.3	93.6	2.4	2.6	1.8	2.6	0.6	0	0	1.3	0.396
Usual activities (%)	83.5	74.4	8.2	18.0	5.9	5.1	1.2	2.6	0.6	0	0.314
Pain / Discomfort (%)	42.9	39.7	37.6	43.6	15.3	12.8	3.5	2.6	0.6	1.3	0.920
Anxiety / Depression (%)	70.0	58.4	24.7	29.5	3.5	9.1	1.7	2.6	0	0	0.047

**Fig. 2:**
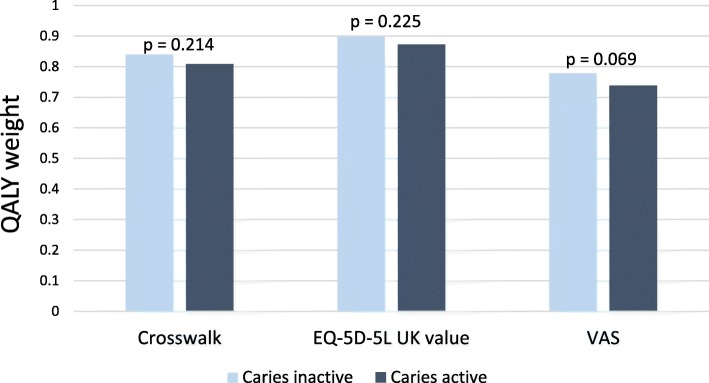
QALY weights of the CI and CA groups estimated by the crosswalk from EQ-5D-5 L to the EQ-5D-3 L, the EQ-5D-5 L UK value, and the VAS

The mean QALY weight tended to be lower in the CA group than in the CI group, implying poorer health among the CA individuals; however, no statistical significance was evident (Fig. 2). Women in the CA group scored themselves significantly higher in the dimension anxiety/depression; no other differences were found between the sexes.

## Discussion

To our knowledge, this is the first study to evaluate an adult population with recurrent caries activity using an established health economic instrument. This study found that the treatment costs are twice as high for CA individuals compared to individuals without caries progression. These findings are in agreement with Söderström et al. [25], further emphasizing the need for health economic evaluations in order to efficiently allocate limited resources. The results show that recurrent caries activity, regardless of age of the individual, is costly in terms of money and time for both the affected individual and society.

Caries and its impact is disease accumulating and this study suggests that the side effects of caries are present even at younger ages. Therefore, more efforts should be put into finding efficient treatment and prevention strategies in younger ages. This study furthermore found that individuals with recurrent caries activity experience more problems related to anxiety and depression measured with EQ-5D-5 L. In line with this, Åkesson et al. found a correlation with caries development and some aspects of mental health [8]. An interview study of caries active adults supports this finding [26]. Finally, our study found no differences regarding QALY weights of the CI and CA groups, which indicates that EQ-5D-5 L may not be able to capture the impact of caries disease.

However, the study population was small and the response rate between the groups was unequal, which may have impacted the outcome. Some differences were found between non-responders and responders, probably due to the small sample of responders. The responders’ distribution in age was skewed as it had a CA group younger than the CI group, which could mean that the individuals with most damage caused by accumulated caries were not included in this study. The inclusion criteria included continuous check-ups, which excluded the caries active individuals absent from check-ups or only coming to see a dentist for acute pain. Previous research has shown regular dental visits improves the oral impacts on daily performance [27]. The CI group in our study was comparable to the norm VAS value in Sweden, which leads to decreased risk for bias according to population sample [28]. Attempts have been done to extract QALY weights from disease specific measurements such as OHIP, but no appropriate translation has been developed [19].

EQ-5D is an often used instrument when performing cost-effectiveness analyses. Such analyses are used by decision makers in order to use scarce resources efficiently. However, since EQ-5D may not be appropriate to estimate the full consequences for patients with caries, further studies are needed that investigate a crosswalk from disease specific instruments to QALY calculations in order to do health economic evaluations in dentistry. Because EQ-5D instrument is the standard questionnaire in health care for economic evaluation, it should not be discarded as an instrument for health economic evaluation in dental research based only on the results from this study. That is, more studies should focus on the usefulness of the instrument in dentistry. To make oral health care more comparable with general health care and to prioritize different treatment strategies in dental care, it is essential to assess the cost-effectiveness of dental interventions.

## Conclusions

This study confirms that the treatment cost of dental caries is high irrespective of age. Young adults tend to have many non-intact teeth and triple treatment costs compared to young individuals free from caries. Reliable instruments for health economic evaluations are needed in dentistry to prioritize treatment methods and to allocate available resources. Further research is needed that investigates suitable instruments for health economic evaluation within dentistry.

## Data Availability

The datasets used and/or analysed during the current study are available from the corresponding author on reasonable request.

## List of abbreviations

a: Approximal
CA: Caries Active group
CI: Caries Inactive group
D2: Decay to Enamel and Dentin Junction
D3: Decay in Dentin
DMFT: Decayed Missed or Filled Tooth
EQ-5D: EuroQol 5 Dimensions
EQ-5D-3 L: EuroQol 5 Dimensions 3 Levels
EQ-5D-5 L: EuroQol 5 Dimensions 5 Levels
HRQoL: Health-Related Quality of Life
OHIP: Oral Health Impact Profile
OHRQoL: Oral Health-Related Quality of Life
QoL: Quality of Life
S: Surface
SEK: Swedish Krona
SF-6D: Short Form 6 dimensions
SG: Standard Gamble
TLV: The Dental and Pharmaceutical Benefits Agency
TTO: Time Trade-Off
VAS: Visual Analogue Scale
